# Trunk Tip Wear in Wild African Savanna Elephants

**DOI:** 10.1093/icb/icaf020

**Published:** 2025-04-29

**Authors:** Olivia Heise, Tabea Pottek, Peter Buss, Lin-Mari de Klerk-Lorist, Lennart Eigen, Susanne Holtze, Guido Fritsch, Frank Göritz, Gudrun Wibbelt, Thomas Hildebrandt, Michael Brecht

**Affiliations:** Bernstein Center for Computational Neuroscience Berlin, Humboldt-Universität zu Berlin, Philippstr. 13, Haus 6, 10115 Berlin, Germany; Bernstein Center for Computational Neuroscience Berlin, Humboldt-Universität zu Berlin, Philippstr. 13, Haus 6, 10115 Berlin, Germany; Veterinary Wildlife Services, South African National Parks, Kruger National Park, 1350 Skukuza, South Africa; State Veterinary Office, Skukuza, South African Department of Agriculture, Land Reform and Rural Development (DALRRD), Kruger National Park, 1350 Skukuza, South Africa; Bernstein Center for Computational Neuroscience Berlin, Humboldt-Universität zu Berlin, Philippstr. 13, Haus 6, 10115 Berlin, Germany; Leibniz Institute for Zoo and Wildlife Research, Alfred-Kowalke-Strasse 17, 10315 Berlin, Germany; Leibniz Institute for Zoo and Wildlife Research, Alfred-Kowalke-Strasse 17, 10315 Berlin, Germany; Leibniz Institute for Zoo and Wildlife Research, Alfred-Kowalke-Strasse 17, 10315 Berlin, Germany; Leibniz Institute for Zoo and Wildlife Research, Alfred-Kowalke-Strasse 17, 10315 Berlin, Germany; Leibniz Institute for Zoo and Wildlife Research, Alfred-Kowalke-Strasse 17, 10315 Berlin, Germany; Bernstein Center for Computational Neuroscience Berlin, Humboldt-Universität zu Berlin, Philippstr. 13, Haus 6, 10115 Berlin, Germany; NeuroCure Cluster of Excellence, Humboldt-Universität zu Berlin, 10117 Berlin, Germany

## Abstract

The anatomy and function of tactile structures, such as vibrissae, are typically studied in captive animals, but we know little about how tactile structures compare between captive and wild animals. We analyzed trunk tip morphology in wild (*n* = 6) and captive (*n* = 6) adult African savanna elephants (*Loxodonta africana*). We found striking differences in both vibrissae and skin structure between the two groups. Wild elephants showed significant vibrissae abrasion, with frontal trunk tip vibrissae often entirely worn down, whereas captive elephants retained proportionally more long vibrissae, particularly along the trunk tip rim. In wild elephants, vibrissae rarely exceeded 1 cm in length, whereas many captive individuals had vibrissae several centimeters long. In contrast, vibrissae inside the nostril—a trunk region not directly exposed to feeding—were similar in length and density between wild and captive elephants. Additionally, trunk tip skin in wild elephants appeared to be worn down to a smooth surface, whereas all captive elephants showed distinct papillary skin structure and folds at the lateral trunk tip opening and nasal septum. These findings suggest that wild elephants experience feeding-related trunk abrasion, leading to significant alterations in both vibrissa structure and skin texture. Our results highlight the importance of studying sensory structures in wild animals to understand sensing in natural environments.

## Introduction

Most of our knowledge about animal anatomy, physiology, and sensory processing comes from studies on captive animals. However, the interactions of captive and wild animals with their environments differ markedly. In captive settings, food is provided and available in abundance, whereas wild animals must adapt to variable resources and actively harvest their food. To understand animal adaptations to natural conditions, it is essential to compare anatomical and behavioral traits between individuals in wild and captive environments. In this study, we explore this issue by analyzing differences between the trunks of captive and wild African savanna elephants (*Loxodonta africana*), hereafter referred to as African elephants.

Elephant facial morphology is a highly derived adaptation from the ancestral mammalian snout. Their trunk, formed by the fusion of the upper lip and nose (Fischer and Trautmann 1987; Schulz et al. 2024), acts as a muscular hydrostat (Kier and Smith 1985) of exceptional complexity and functionality. In Asian elephants, the trunk contains approximately 90,000 muscle fascicles (Longren et al. 2023) and around 400,000 tactile axons, making it both a powerful manipulative and highly sensitive sensory organ (Purkart et al. 2022). African elephants rely heavily on trunk use during feeding. Despite their large body size and correspondingly low metabolic rate, they must consume vast amounts of food daily. Their mixed diet of shrubs, trees, and grasses (Douglas-Hamilton 1972), combined with en-passant feeding behavior—gathering food while walking—maximizes foraging efficiency. Our personal observations of elephants in Kruger National Park support the idea that elephants spend the majority of their time feeding.

Tactile specialization in the elephant trunk includes prominent black vibrissae, especially dense at the tip (Rasmussen and Munger 1996; Deiringer et al. 2023). A baby African elephant was found to have around 1200 trunk vibrissae. In zoo elephants, vibrissae show laterally asymmetric abrasion, consistent with lateralized trunk use (Deiringer et al. 2023). Roughly half of all elephants are left-trunked, and the other half are right-trunked (Giljov et al. 2017; Lefeuvre et al. 2022). Additionally, the anatomy of the lower lip vibrissae and trunk usage during feeding suggest elephants insert food laterally into the mouth—a deviation from the frontal insertion seen in most other mammals (Yildiz et al. 2024).

The skin of the elephant trunk is also quite peculiar. It is thicker than even the carapace of armadillos (Schulz et al. 2022) and is marked by dense folds and wrinkles, particularly toward the distal trunk (Schulz et al. 2024). These folds appear early in development and are likely integral to the trunk’s flexibility and multifunctionality. In this study, we investigate how trunk vibrissae and skin differ between captive and wild African elephants. We also compare the condition of the trunk skin across environments. We observe marked differences, including signs of extreme trunk wear in wild elephants, that suggest significant environmental impact on trunk morphology.

Our hypothesis is that environmental conditions and feeding behaviors in the wild lead to measurable morphological differences in trunk anatomy compared to captivity. By characterizing these differences, we aim to provide new insights into how the elephant trunk adapts to different ecological pressures. This work has broader implications for understanding how specialized sensory structures evolve and function in response to environmental demands.

## Materials and methods

### Animal specimens and photography

All animals included were African savanna elephants (*L. africana*). Photographs were taken at the lab in Berlin and in Kruger National Park with a handheld Sony α 7R III camera or a Sony α 7S II with a Sony FE 16–35 mm F2.8 GM E-Mount objective or a Sony FE 90 Mm/2.8 Macro G OSS objective. Apart from adjustments to brightness and contrast using Adobe Photoshop (Adobe Systems, San Jose, California, USA) no further image alterations were made.

#### Wild elephants

All photographs of wild elephant trunks were taken in Kruger National Park. Images were obtained either from post-mortem African elephant bulls (*n* = 4) or during short-term anesthesia in the field (*n* = 2) as part of unrelated health check-ups. For necropsy analysis, elephant age was estimated by examining the teeth in the lower jaw, following the method described by Laws (1966). The wild elephants included in this study were numbered sequentially, based on the order of inspection, followed by the year, as detailed in Table 1. The necropsy material was obtained from elephants classified as damage-causing animals, which were harvested to mitigate human-elephant conflict in Kruger National Park. All photographs were taken in accordance with the SANS Parks research application SS1387, and collected material followed the guidelines of the respective collection permit SKZ177.

**Table 1:** Overview of the African savanna elephant zoo and wild specimens.

Name	Zoo/Wild	Species	Sex	Age (years)	Origin
Indra	Zoo	*L. africana*	F	34	Elefantenhof Platschow, Germany
Linda	Zoo	*L. africana*	F	34	Zoo Poznan, Poland
Bibi	Zoo	*L. africana*	F	41	Zoo Aalborg, Denmark
Nyioka	Zoo	*L. africana*	F	44	Knuthenborg Safaripark, Denmark
Ali	Zoo	*L. africana*	M	23	Opel-Zoo Kronberg, Germany
Kibo	Zoo	*L. africana*	M	45	Valencia—Bioparc Valencia, Spain
Bull 2_23	Wild	*L. africana*	M	n.a.	Kruger National Park, South Africa
Bull 3_23	Wild	*L. africana*	M	n.a.	Kruger National Park, South Africa
Bull 4_23	Wild	*L. africana*	M	25	Kruger National Park, South Africa
Bull 1_24	Wild	*L. africana*	M	22	Kruger National Park, South Africa
Bull 2_24	Wild	*L. africana*	M	27	Kruger National Park, South Africa
Bull 3_24	Wild	*L. africana*	M	39	Kruger National Park, South Africa

Note that in the investigation of some wild specimens, there was not enough time to perform dental aging procedures.

#### Zoo elephants

Photographs were also taken from necropsy samples of captive African zoo elephant trunks. All zoo elephants included in the study died of natural causes or were euthanized by experienced zoo veterinarians for welfare reasons because of serious health complications. Table 1 gives an overview of the specimens of African elephants (*L. africana*), along with the age, sex, and origin.

### Vibrissa length measurements

A preliminary inspection showed the most marked vibrissa differences between wild and zoo elephants related to the trunk tip. According to a qualitative assessment, vibrissae of the mouth region and the more proximal trunk regions were less consistently different between wild and zoo elephants. We focused our quantitative analysis on the African elephant trunk tip. To this end, we analyzed frontal photographs of the trunk tip using Adobe Photoshop. Photographs were scaled according to a scalebar placed in the initial photograph, and we used the ruler tool to measure vibrissa length. Vibrissa length was measured as distance from vibrissa origin to vibrissa tip; this measure omits vibrissa bending. We also counted missing/zero-lengths vibrissae. Such zero-length vibrissae were counted if (i) the pit, from which the elephant vibrissa originate, could be identified; (ii) a dark spot or a darkening inside this pit indicated an empty vibrissa origin; (iii) if the zero-length vibrissa was arranged in the vibrissa row pattern characteristic for elephant trunk vibrissae. All ambiguous cases, where zero-length vibrissae could not be identified according to the above three criteria outlined above, were excluded from the analysis. Consequently, our findings likely underestimate the total number of zero-length vibrissae. To facilitate systematic quantification, we divided the frontal trunk tip into distinct counting zones, as shown in Fig. 2.

### MicroCT scans

Three trunk tip skin samples of approximately 3 cm^3^ were cut out laterally of the dorsal and ventral trunk finger, and the most lateral part of Bull 1_24 trunk tip. Samples were stained using Lugol’s solution. Scanning and Data Visualization: MicroCT scans were acquired at Museum für Naturkunde Berlin, Germany, through a YXLON FF85 CT system (YXLON International GmbH, Hamburg, Germany) equipped with a Perkin Elmer Y Panel 4343 CT detector and 190 kV nano focus transmission tube. Transmission images were stepwise obtained over 360° rotation, with 1 s exposure. Tomographic reconstruction was performed using the built-in YXLON reconstruction software with standardized settings. 3D images of the samples were rendered using an extended version of Amira.

## Results

### Trunk vibrissae and skin differ between captive and wild African elephants

**Fig. 1:**
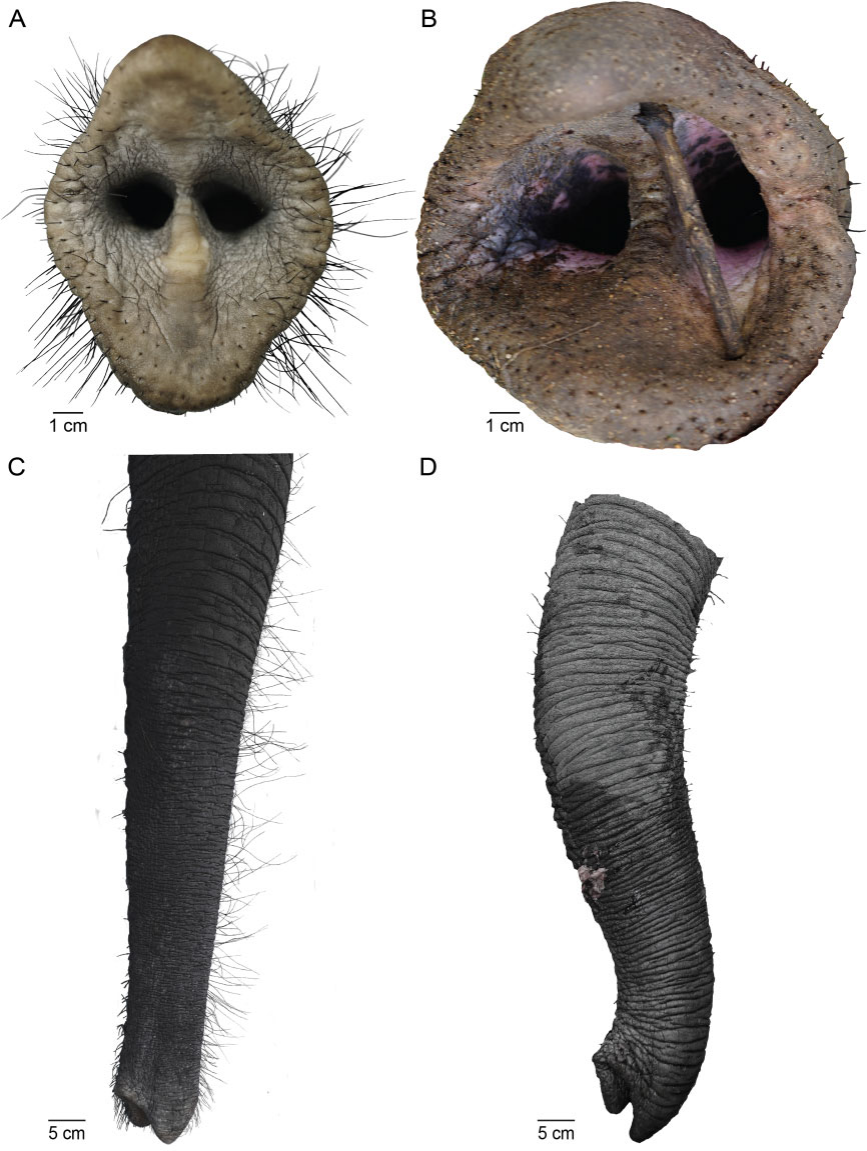
Trunk tips and trunks of captive and wild African savanna elephants. (A) Trunk tip of African savanna elephant Bibi, a 41-year-old female elephant from Aalborg Zoo. Note the numerous and long vibrissae. (B) Trunk tip of wild African savanna elephant, an elephant bull from Kruger National Park. Note the absence of long vibrissae. (C) Distal trunk of Zoo elephant Bibi. Note the numerous and long vibrissae. (D) Distal trunk of a wild African savanna elephant bull 3_24. Note the absence of vibrissae.

**Fig. 2:**
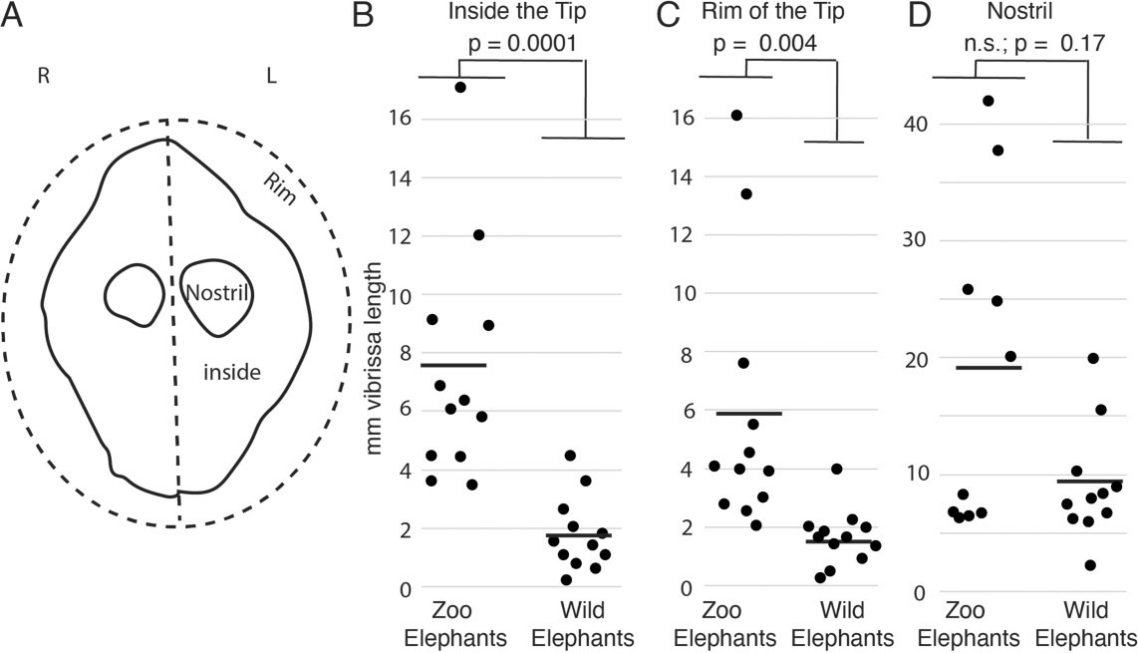
Quantification of trunk tip vibrissa length differences between captive and wild African savanna elephants. (A) Schematic of elephant trunk tip with the different areas in which we quantified vibrissa length (left and right side, rim of the tip, inside the tip, nostril). (B) Vibrissa length inside the trunk tip of 6 zoo and 6 wild African savanna elephants; vibrissa length was quantified separately for left and right hemi-trunk-tip. Each dot corresponds to the average vibrissa length in hemi-trunk tips of individual elephants. Thick lines represent averages across captive and wild elephants, respectively. Clearly recognizable vibrissa origins with fully abrased vibrissae, were counted as zero-length vibrissae. *P*-value refers to unpaired *t*-tests between captive and wild elephants. (C) Vibrissa length along the trunk tip of captive and wild elephants; conventions as in (B). (D) Vibrissa length in the nostril of captive and wild elephants, the length differences are not significant; conventions as in (B).

The key result of our study is that the trunk tips of captive and wild African savanna elephants differ in regard to whisker and skin abrasion. We show these differences, for example, trunk tips in Fig. 1, and quantitatively compare trunks across animals in Fig. 2. In Fig. 1A, we show the trunk tip of zoo elephant Bibi, an adult female. As described previously, the trunk tip is covered with prominent black vibrissae (Deiringer et al. 2023). The trunk tip of a wild African elephant bull, shown in Fig. 1B, looks strikingly different. The trunk tip vibrissae are largely abrased, the few remaining vibrissae are short. Similar vibrissa differences are apparent when inspecting the entire distal trunk. In the captive elephant Bibi, numerous long vibrissae are seen (Fig. 1C), while the distal trunk of another wild bull (Fig. 1D) shows few and short vibrissae. We conclude that vibrissae of the distal trunk and the trunk tip are present in captive but are abrased in wild African elephants.

### Quantitative assessment of trunk tip vibrissa length in captive and wild African elephants

We sought to quantify differences between captive and wild elephants. To this end, we focused on the trunk tip, which we divided into six areas (Fig. 2A; left vs. right, rim of the trunk tip, inside the trunk tip, and nostril). We quantified origin to tip vibrissa length (ignoring bending) in each of these zones. We observed approximately 3.5-fold shorter vibrissa length inside the trunk tip of wild compared to zoo elephants (Fig. 2B), and this difference was highly significant (*P* < 0.0001; unpaired *t*-test). Similarly, we observed approximately 4-fold shorter vibrissa length along the rim of the trunk tip for wild compared to zoo elephants (Fig. 2C), and this difference was also significant (*P* < 0.004; unpaired *t*-test). In contrast, vibrissa length was not significantly different in the nostrils of wild and zoo elephants (Fig. 2D). We conclude that wild African savanna elephants encounter significant vibrissa abrasion.

### Trunk tip skin differences between captive and wild African elephants

**Fig. 3:**
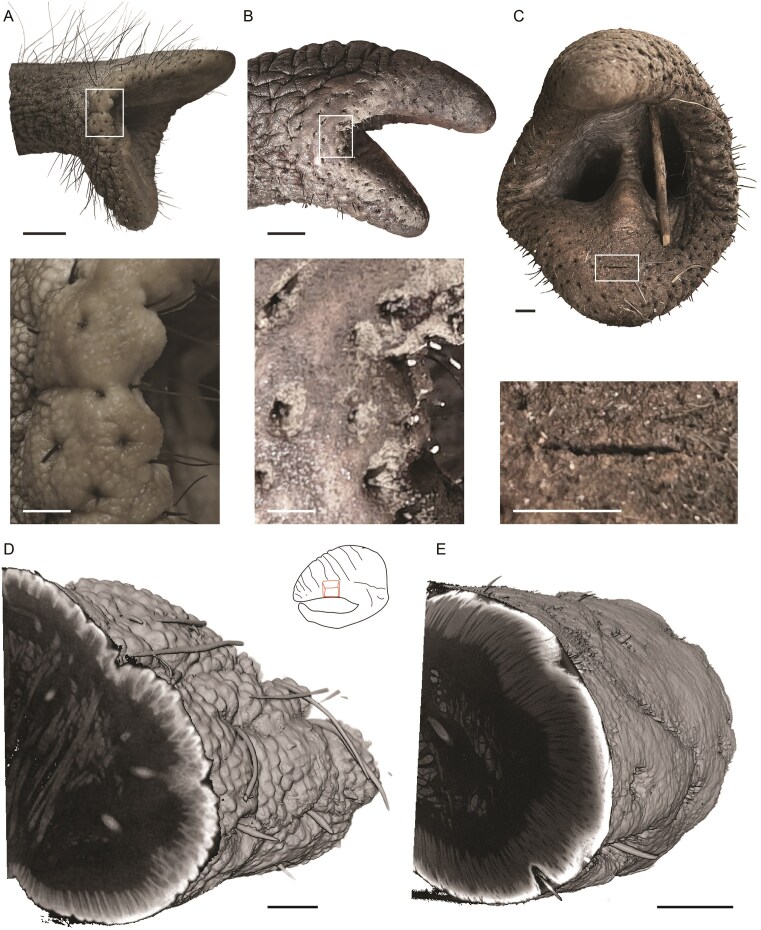
Trunk tip skin abrasion is more pronounced in wild than in captive African savanna elephants. (A) Upper, side view of the trunk tip of African savanna elephant Bibi, a 41-year-old female elephant from Aalborg Zoo. Lower, high magnification view (white box in the upper image) of skin folds in the corner of the trunk tip opening. (B) Upper, side view of the trunk tip of a wild African savanna elephant, an elephant bull from Kruger National Park. Lower, high magnification view (white box in the upper image) of largely abrased skin folds are in the corner of the trunk tip opening. (C) Upper, frontal view of the trunk tip of wild African savanna elephant from Kruger National Park. Lower, high magnification view (white box in the upper image) of a skin crack (center of the image) in the trunk tip of the wild elephant. (D) Left, volume rendering of microCT scan of an iodine-stained trunk tip piece from an African Zoo elephant. Note the hair and the papillary structure of skin visible both in section and the surface of the sample. Right, schematic of the trunk tip, in red the cut-out section. (E) Volume rendering of microCT volume image of an iodine-stained trunk tip piece from a wild African elephant. Note the smooth surface and the absence of the papillary skin structure visible both in section and the surface of the sample. The trunk tip sample comes from the same trunk position as indicated in (D) (upper). Scalebars in upper A, B 50 mm; lower panels A, B, C 10 mm; C, D, E 10 mm.

Differences between wild and captive African elephants were not restricted to trunk vibrissae. Specifically, the skin of wild elephants often looked like it had been treated with sandpaper. Zoo elephants were observed to have prominent skinfolds in the corner of the trunk tip opening, as seen in Fig. 3A. In contrast, in the corner of the trunk tip opening of wild elephants, such skin folds were often less apparent and appeared to be abrased (Fig. 3B). We also observed cracks in the trunk tip skin of wild individuals (Fig. 3C). We also obtained microCT scans of trunk tip pieces from wild and zoo African elephants. In African zoo elephant trunks, we consistently observed a papillary skin structure apparent both on the trunk surface and in image sections through the trunk sample (Fig. 3D). In wild elephants, however, the papillary skin structure was abrased (Fig. 3E). Our observations on skin abrasion reinforce the view that the trunk tips of wild elephants experience significant wear.

## Discussion

We obtained trunk samples of captive and wild African elephants and found these trunks to be remarkably different. While captive African elephants have prominent trunk vibrissae and trunk tip skin folds, wild African elephant vibrissae are largely abraded, and trunk tip skin papillae and folds were worn down. We discuss the reasons for these differences and the implications of these findings.

### Trunks and trunk tips of captive and wild African elephants differ

We were able to obtain a large number of trunk tips from zoo and wild African elephants in the last decades. All data presented here refer to adult individuals. We would expect our results might differ if the sample included elephant calves, which—in zoo elephants—show much less vibrissa abrasion than adult animals (Deiringer et al. 2023). Our observations on vibrissa patterns in captive African elephants are consistent with and support the findings of Deiringer et al. (2023). African zoo elephants show prominent black trunk tip vibrissae that often display lateralized abrasion. Additionally, the trunk tip features shorter and denser vibrissae compared to more proximal trunk regions. The trunk tips of wild African elephants have vibrissae either worn down to a short length or absent altogether. Importantly, the vibrissa length differences were highly significant between captive and wild individuals. The nostril vibrissae were not different between wild and zoo elephants. Unlike the rim and the inside the tip vibrissae, the nostril vibrissae are not in contact with food items. This observation supports the fact that differences between wild and zoo elephants might arise from differences in food collection. The trunk tip skin also differs between captive and wild African elephants. Specifically, the prominent papillary structure of the skin and the skin folds that are seen on the trunk tip of almost every zoo elephant are worn down in wild elephants.

### Trunk wear in captive and wild African elephants

Vibrissa abrasion is present in both wild and captive elephants; such abrasion appears to be much more pronounced in wild elephants, however. The data suggest that the differences in trunk tips between captive and wild individuals are a result of the extreme wear experienced by wild elephants. This is both a mechanically and behaviorally plausible explanation. Already, our earlier evidence from zoo elephants pointed to use-dependent abrasion of trunk vibrissae (Deiringer et al. 2023). Specifically, we observed vibrissa differences between the left and right trunk tip side and observed longer less abraded trunk vibrissae in elephant calves compared to adult animals (Deiringer et al. 2023). Our own informal observations on feeding behavior of captive and wild elephants are in line with the idea that wild elephants experience more wear. Zoo elephants typically have circumscribed feeding hours and are usually served easily apprehensible food items such as hay. Elephants in the wild are almost constantly engaged in feeding behaviors (up to 18 h a day, Lefeuvre et al. 2020), they sleep very little (Gravett et al. 2017), the food they take up is of mixed quality, and many of the forage plants carry defensive thorns and hardenings. Accordingly, elephants can be observed pulling branches of Acacias with often large thorns through the distal end of the trunk wrapped around the branch prior to feeding on them. So far, our work is restricted to elephant bulls from the wild, and future work should also include wild elephant cows. Our study adds to the growing recognition of the need to study vibrissae under natural conditions. Similarly, naturalistic stimulus paradigms such as hydro-dynamic trail following in seals (Dehnhardt et al. 2001) have greatly contributed to our understanding of vibrissa function. In summary, we suggest that the acquisition of large food quantities in wild elephants leads to extreme trunk tip wear. Zoo elephants experience less wear in contrast, because food is provided.

### Is trunk wear relevant for elephant survival in the field?

The vibrissa and skin abrasion patterns documented in our study indicate wear of elephant trunk tips in the field. We also documented trunk skin breakage, i.e., extents of skin damage that expose the elephants to the entry of pathogens. It remains to be determined if such trunk wear diminishes trunk functionality and is relevant for elephant survival. In particular, it is of interest to study the state of the trunk tip in aged elephants in the field, which presumably have a decreased capacity to regenerate skin to compensate for trunk wear. Classically, attention has focused on the wear down of elephant teeth, and there is widespread agreement that tooth wear and tear might limit elephant life expectancy (Laws 1966).

### Sensory implications of vibrissa abrasion and elephant vibrissa growth

We describe a more than twofold length difference in trunk tip vibrissae of zoo and wild African elephants. Vibrissa length is a major mechanical determinant of vibrissa function, and we suggest that abrasion will affect elephant vibrissa sensing. Likely, the effects of enhanced vibrissa abrasion will be larger for longer vibrissae than for the very short trunk tip vibrissae. In our earlier work, we observed that no dual vibrissae appear in elephant vibrissa follicles (Deiringer et al. 2023). Classic studies on whisker function have investigated both captive (Vincent 1912) and wild animals (Pocock 1914). To our knowledge, however, the possibility that vibrissae might differ between wild and captive animals has found relatively little consideration in the literature. Earlier authors have addressed the question, how ambient conditions may affect vibrissa function, however (Dehnhardt et al. 1998). In rodents, many vibrissa follicles carry dual vibrissae, and the old vibrissa falls out, when the new vibrissa grows to the full length (Maier and Brecht 2018). Thus, rodent vibrissa replacement leads to relatively constant length vibrissae. We suggest that elephant vibrissa length might be determined by a steady balance of vibrissa growth and abrasion instead. Besides a better understanding of elephant vibrissa replacement/growth, we also need further work on the mechanisms of vibrissa wear/breakage. Interestingly, we found that vibrissae located within the nostril are nearly double, and in some cases almost triple, the length of those situated on the rim or tip of the trunk. This pattern was evident not only in wild African elephants but also in captive individuals, suggesting a conserved trait rather than an environmentally induced difference. The increased length of intranasal vibrissae may reflect their unique sensory role. Unlike the vibrissae on the tip or rim, which are directly involved in tactile exploration and object manipulation, those within the nostril may serve to detect airflow, particulate matter, or internal mechanical deformation during trunk use.

## Conclusion

Trunk tips of zoo and wild African elephants differ. It appears the challenges of food acquisition in the wild lead to considerable trunk wear. Our work shows that vibrissae can look dramatically different in captive vs. wild animals. Specifically, the mechanical interactions form vibrissae with food items shape different vibrissae patterns in captive and wild animals. The vast majority of work on vibrissae comes from captive animals, however. An implication of these findings is therefore that more field work on vibrissa function needs to be done in vibrissa research.

## Author contributions

Conceptualization, O.H. and M.B.; Methodology & Materials, O.H., T.P., P.B., L.M.K.L., L.E., S.H., G.F., F.G., G.W., T.H., and M.B.; Investigation, O.H., T.P., P.B., L.M.K.L., L.E., S.H., G.F., F.G., G.W., T.H., and M.B.; Formal Analysis, O.H. and M.B.; Writing O.H., T.H., and M.B.; Supervision, M.B.; Funding Acquisition, M.B.

## Data Availability

All relevant data are included in the article. We share additional elephant material upon request to M.B.

## Notes

“From the symposium “Fleshing it out: recent advances in form, function and motor control of biological hydrostats” presented at the annual meeting of the Society for Integrative and Comparative Biology, January 3-7th , 2025.”
